# Rapid transcriptional and metabolic regulation of the deacclimation process in cold acclimated *Arabidopsis thaliana*

**DOI:** 10.1186/s12864-017-4126-3

**Published:** 2017-09-16

**Authors:** Majken Pagter, Jessica Alpers, Alexander Erban, Joachim Kopka, Ellen Zuther, Dirk K. Hincha

**Affiliations:** 1 Max-Planck-Institut für Molekulare Pflanzenphysiologie, Am Mühlenberg 1, D-14476 Potsdam, Germany; 2 Present address: Chemistry and Bioscience, Aalborg University, Fredrik Bajers Vej 7H, DK-9220 Aalborg East, Denmark

**Keywords:** *Arabidopsis thaliana*, Cold acclimation, Deacclimation, Gene expression, Metabolomics, Transcriptomics

## Abstract

**Background:**

During low temperature exposure, temperate plant species increase their freezing tolerance in a process termed cold acclimation. This is accompanied by dampened oscillations of circadian clock genes and disrupted oscillations of output genes and metabolites. During deacclimation in response to warm temperatures, cold acclimated plants lose freezing tolerance and resume growth and development. While considerable effort has been directed toward understanding the molecular and metabolic basis of cold acclimation, much less information is available about the regulation of deacclimation.

**Results:**

We report metabolic (gas chromatography-mass spectrometry) and transcriptional (microarrays, quantitative RT-PCR) responses underlying deacclimation during the first 24 h after a shift of *Arabidopsis thaliana* (Columbia-0) plants cold acclimated at 4 °C back to warm temperature (20 °C). The data reveal a faster response of the transcriptome than of the metabolome and provide evidence for tightly regulated temporal responses at both levels. Metabolically, deacclimation is associated with decreasing contents of sugars, amino acids, glycolytic and TCA cycle intermediates, indicating an increased need for carbon sources and respiratory energy production for the activation of growth. The early phase of deacclimation also involves extensive down-regulation of protein synthesis and changes in the metabolism of lipids and cell wall components. Hormonal regulation appears particularly important during deacclimation, with extensive changes in the expression of genes related to auxin, gibberellin, brassinosteroid, jasmonate and ethylene metabolism. Members of several transcription factor families that control fundamental aspects of morphogenesis and development are significantly regulated during deacclimation, emphasizing that loss of freezing tolerance and growth resumption are transcriptionally highly interrelated processes. Expression patterns of some clock oscillator components resembled those under warm conditions, indicating at least partial re-activation of the circadian clock during deacclimation.

**Conclusions:**

This study provides the first combined metabolomic and transcriptomic analysis of the regulation of deacclimation in cold acclimated plants. The data indicate cascades of rapidly regulated genes and metabolites that underlie the developmental switch resulting in reduced freezing tolerance and the resumption of growth. They constitute a large-scale dataset of genes, metabolites and pathways that are crucial during the initial phase of deacclimation. The data will be an important reference for further analyses of this and other important but under-researched stress deacclimation processes.

**Electronic supplementary material:**

The online version of this article (10.1186/s12864-017-4126-3) contains supplementary material, which is available to authorized users.

## Background

Plants native to temperate and boreal climates show natural low temperature acclimation during fall in preparation for winter frost. This process is termed cold acclimation. In spring, plants lose the freezing tolerance acquired during acclimation by deacclimation while they resume growth and development [1]. Cold acclimation has been extensively studied and the low temperature acclimation response is a multigenic, quantitative trait involving massive re-programming of the transcriptome and metabolome (see [2–4] for reviews). Much of the effort in cold acclimation research has focused on the regulation of cold-responsive gene expression and transcript profiling data suggest the induction of multiple transcriptional pathways [5]. Currently, the best understood cold acclimation signalling pathway depends on the C-REPEAT BINDING FACTOR (CBF) family of APETALA2 (AP2) type transcriptional activators. *CBF* genes appear to be ubiquitous in plants and are almost always present in multiple copies [4–6].

While considerable effort has been directed toward understanding how plants cold acclimate and adapt to low temperature, deacclimation and the persistence of the acclimated state under warm conditions have not attracted much attention. However, the timing and rate of deacclimation may be key determinants of survival during late winter and early spring [7, 8]. The topical interest in climate change further emphasizes the importance of increased knowledge on deacclimation in plants. Global climate models predict an increase in the mean surface air temperature and in the frequency and severity of erratic temperature events [9]. Hence, winters in temperate regions are becoming progressively milder and temperature patterns are becoming increasingly irregular. This increases the frequency of warm spells that may cause premature deacclimation, thereby increasing the risk of subsequent freezing injury [8, 10]. Additionally, shifting phenological patterns, such as an earlier start of the growth season and earlier flowering [11, 12], consistent with climate warming, can increase the risk of tissue damage by subsequent frost. The likelihood of such scenarios is typically high during early spring [13].

Deacclimation, quantified as a reduction in freezing tolerance under controlled conditions, is a fast process leading to substantial decreases in freezing tolerance within a few days. The exact extent and kinetics, however, depend on deacclimation temperature, plant species and genotype [14–17]. A limited number of studies focusing on the metabolic and molecular mechanisms underlying deacclimation have been reported. Most extensively documented is the association between decreasing concentrations of specific soluble carbohydrates and loss of freezing tolerance [15, 17–19]. In addition, two transcriptomic studies on deacclimation have been published [14, 20]. Both were conducted using *Arabidopsis thaliana* and showed that the abundance of transcripts of almost all cold induced genes is strongly reduced during deacclimation. Using the same species it was recently shown that deacclimation is a tightly regulated process, which includes coordination of the CBF regulon [17] and the plastidic antioxidant system [21]. The CBF signal transduction pathway has also been implicated in deacclimation of *Betula pendula*, where members of the CBF regulon are down-regulated only after prolonged exposure to warm temperatures, presumably enabling plants to maintain freezing tolerance during short warm spells [22]. However, except for the CBFs, regulators of deacclimation remain largely unknown. Time dependence of transcriptional [14, 17] and metabolic [17, 18] changes during deacclimation has been recognized, but mostly on a time scale of days (transcriptional changes) or days to weeks (metabolic changes), which is insufficient to recognize fast regulatory responses.

Therefore, we have investigated the initial metabolic (gas chromatography-mass spectrometry (GC-MS)) and transcriptional (microarrays, qRT-PCR) responses during the first 24 h after a shift of plants cold acclimated for three days at 4 °C to 20 °C day/18 °C night temperatures. Our analysis reveals that the transcriptome responds more rapidly than the metabolome and that deacclimation involves tightly regulated transcriptional and metabolic responses.

## Methods

### Plant material and growth conditions

*Arabidopsis thaliana* accession Columbia-0 (Col-0) was grown in soil in a greenhouse at 8 h day length with light supplementation to reach at least 200 μmol m^−2^ s^−1^ and a temperature of 20 °C during the day, 18 °C during the night. After three weeks of growth under these conditions plants were transferred to long days (16 h light/8 h dark), but otherwise similar growth conditions.

For cold acclimation, 4 weeks old plants were transferred to a 4 °C growth cabinet at 16 h day length with 90 μmol m^−2^ s^−1^ for three days, following our previously established cold acclimation protocol [23, 24]. For deacclimation, cold acclimated plants were transferred back to the greenhouse at 20 °C/18 °C day/night temperature and at least 200 μmol m^−2^ s^−1^ light as described previously [17]. Complete rosettes were harvested from 10 individual replicate plants, immediately frozen in liquid nitrogen, stored at −80 °C and later powdered using a ball mill (Retsch, Haan, Germany). The experiment was performed in three independent biological replicates.

### qRT-PCR analysis of the expression of genes encoding transcription factors

Transcript levels of genes encoding transcription factors (TFs) were analyzed in non-acclimated and cold acclimated plants and plants deacclimated at 20 °C for 2 h, 4 h, 6 h, 12 h or 24 h using a real-time qRT-PCR platform. The TF platform contains 1880 primer pairs in five 384-well plates to determine the abundance of transcripts from the majority of genes encoding TFs in *Arabidopsis* [25–27]. The analysis included three independent biological replicates, giving a total of 21 samples.

Total RNA was isolated from a pool of rosettes from 10 different plants for each sample, using Trizol reagent (Invitrogen, Carlsbad, CA) and DNase treated with RapidOut DNA Removal Kit (Thermo Scientific, Waltham, MA). RNA quantity and quality were determined using a Nanodrop spectrophotomer (Nanodrop Technologies, Wilmington, DE) and gel electrophoresis. Quantitative PCR with intron-specific primers [28] was used to ascertain the absence of genomic DNA. cDNA was synthesized with SuperScript III reverse-transcriptase (Invitrogen, Carlsbad, CA) and oligo-dT20 primers. cDNA quality was checked using primers amplifying 3′ and 5′ regions of *GAPDH* (At1g13440) [28]. qRT-PCR was performed as described [29]. Ct values for TF genes were normalized by subtracting the mean Ct of four reference genes *GAPDH*, *PDF2*, *Actin2* and *EXPRS* that were included on each plate [25].

A first step data analysis performed by Principal Components Analysis (PCA) applied to the normalized Ct values indicated that one replicate in each of two samples were outliers. Hence, these replicates were excluded from further analysis. Finally, only genes with at least two replicate Ct values per time point and with Ct values determined in ≥75% of all samples were considered for further analysis. This resulted in 1462 TF genes whose expression was analyzed in detail. A complete list of all respective expression values can be found in Additional file 1.

The log_2_ transformed relative expression values (i.e. the Ct values normalized to the expression of the reference genes) from the qRT-PCR measurements were analyzed using ANOVA type I SS with correction for multiple testing using the Benjamini-Hochberg [30] method (false discovery rate (FDR) *P* < 0.01) by comparing all treatments against each other in R. Fold change was calculated to cold acclimated samples and only genes with a log_2_ fold ratio greater than 1 or lower than −1 were considered for the subsequent t-tests (FDR *P* < 0.05) where we compared non-acclimated and deacclimated samples to cold acclimated samples. Enrichment of differentially expressed TF genes in particular TF families following different durations of deacclimation was tested for significance by applying Fisher tests with a Bonferroni correction for multiple tests [29].

### Global transcript profiling

Total RNA was isolated as described above and quality was additionally assessed using a bioanalyzer (Agilent, Santa Clara, CA). cRNA synthesis, labelling, hybridization onto Affymetrix Genechip *Arabidopsis* Gene 1.0 ST Arrays and scanning was performed at ATLAS Biolabs GmbH (Berlin, Germany). The total number of RNA samples processed was 21, comprising three independent biological replicates of each of the seven conditions described above.

The raw *Arabidopsis* Gene 1.0 ST intensities were imported into the RobiNA software [31] to perform quality assessment and data normalization and to identify genes differentially regulated between cold acclimated plants and plants subjected to different durations of deacclimation. Data normalization was performed using the RMA method. Statistical analysis of pair-wise differential gene expression between cold acclimated and deacclimated or non-acclimated plants was carried out using a linear model-based approach, applying a 0.05 cut-off for *P*-values after Benjamini-Hochberg correction for multiple testing [30] and log_2_ fold ratio greater than 1 or lower than −1. A list of all genes that were significantly regulated at least at one timepoint during deacclimation, or between cold acclimated and non-acclimated plants is provided in Additional file 2.

### GC-MS metabolite profiling

Polar metabolites from non-acclimated and cold acclimated plants and plants deacclimated for 2 h, 4 h, 6 h, 12 h or 24 h were extracted and processed as described previously [32]. Gas chromatography coupled to electron impact ionization-time of flight-mass spectrometry (GC/EI-TOF-MS) was performed and metabolites were identified as described previously [33]. Metabolite intensities were normalized to sample fresh weight and the internal standard ^13^C_6_-sorbitol. In all cases, six biological replicates, two from each of the three independent experiments, were included.

A total of 168 polar metabolites were identified, including known and yet unknown compounds, archived by the Golm Metabolome Database [34]. Three metabolites that were detected in less than 50% of the non-acclimated and cold acclimated samples or in less than half of the total samples, and where the absence was not related to acclimation state, were excluded from the dataset. In addition, sucrose was removed from the dataset. Sucrose was present in our analyses but in most samples saturated beyond its upper detection limit. A complete list of the normalized metabolite levels can be found in Additional file 3.

Metabolite levels were log_10_ transformed and the statistical significance of differences in metabolite pool sizes was tested by ANOVA at *P* < 0.05 with Benjamini-Hochberg correction for multiple testing by comparing all conditions against each other. Data were log_10_ transformed only for this analysis. Hence, the data presented in Fig. 4 are normalized responses that represent relative metabolite abundance measures.

### Further data analysis methods

Principal Components Analysis (PCA) was performed using the *pcaMethods* package in R [35]. For the microarray data, significant enrichment of functional categories of the MapMan annotation bins among significantly differentially expressed genes was tested by applying Fisher tests with a Benjamin-Hochberg correction for multiple testing using PageMan [36].

Hierarchical clustering of statistically significantly changed metabolite or transcript pool sizes using a Euclidian distance as the distance measure, and the average linkage to define similarity between the clusters was performed with the MultiExperiment Viewer software [37].

## Results

### Effects of deacclimation on transcript and metabolite abundance of cold acclimated plants

**Fig. 1:**
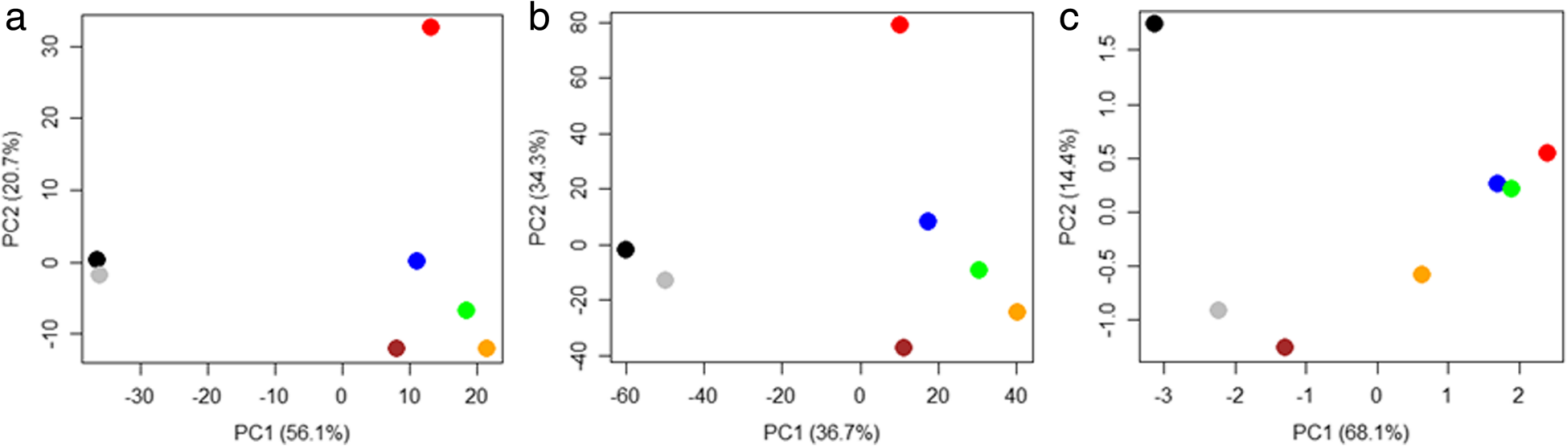
Score plots from principal components analysis (PCA). PCA was applied to **a** qRT-PCR, **b** microarray and **c** GC-MS metabolite profiling data sets. Plants were non-acclimated (black) or cold acclimated at 4 °C for 3 days (red). Cold acclimated plants were then deacclimated at 20 °C for 2 h (blue), 4 h (green), 6 h (orange), 12 h (brown) or 24 h (grey)

As an initial step in the data analysis, PCA was used to identify the largest variance components in the GC-MS metabolite data, and the global (whole-genome microarray) and targeted (qRT-PCR of transcript levels of 1880 TF genes) transcript data. As evident from Fig. 1, both transcript and metabolite data follow a circular trajectory. For the transcripts, principal component 1 (PC1) separated profiles from cold acclimated plants and plants deacclimated for 2 h, 4 h, 6 h and 12 h from those of non-acclimated plants and plants deacclimated for 24 h. It explained 56.1% and 36.7% of the variance for the qRT-PCR and microarray data, respectively. PC2 separated profiles of cold-acclimated plants from non-acclimated and deacclimated plants and explained 20.7% and 34.3% of the variation in the two datasets. For the metabolites, acclimation state was also the dominant source of variance underlying PC1 (68.1% of total variance). The samples from non-acclimated and cold acclimated plants were distributed furthest apart, while samples from deacclimated plants followed an order from cold acclimated to 2 h, 4 h, 6 h, 12 h, 24 h of deacclimation to non-acclimated. This indicates that changes in metabolite content during deacclimation followed a strictly controlled time-course. PC2 of the metabolite data set distinguished samples from plants deacclimated for 6 h, 12 h and 24 h from samples from non-acclimated and cold acclimated plants and from plants deacclimated for 2 h and 4 h (14.4% of total variance).

According to the PCA analyses the responses of cold acclimated plants to warm conditions followed different kinetics at the transcript compared to the metabolite levels. In the case of transcripts, profiles of cold acclimated plants were markedly separated already from those of plants deacclimated for 2 h, implying that even a short exposure to warm temperatures was sufficient to induce a significant transcriptional response. In contrast, metabolite profiles from plants deacclimated for 2 h and 4 h remained similar to profiles from cold acclimated plants. Also, profiles from non-acclimated plants and from plants deacclimated for 24 h co-clustered in the case of transcripts encoding TFs (Fig. 1a) and to a lesser extent at the global transcript level (Fig. 1b), while this was not observed for the metabolite data (Fig. 1c).

### Transcriptional regulation during deacclimation

**Fig. 2:**
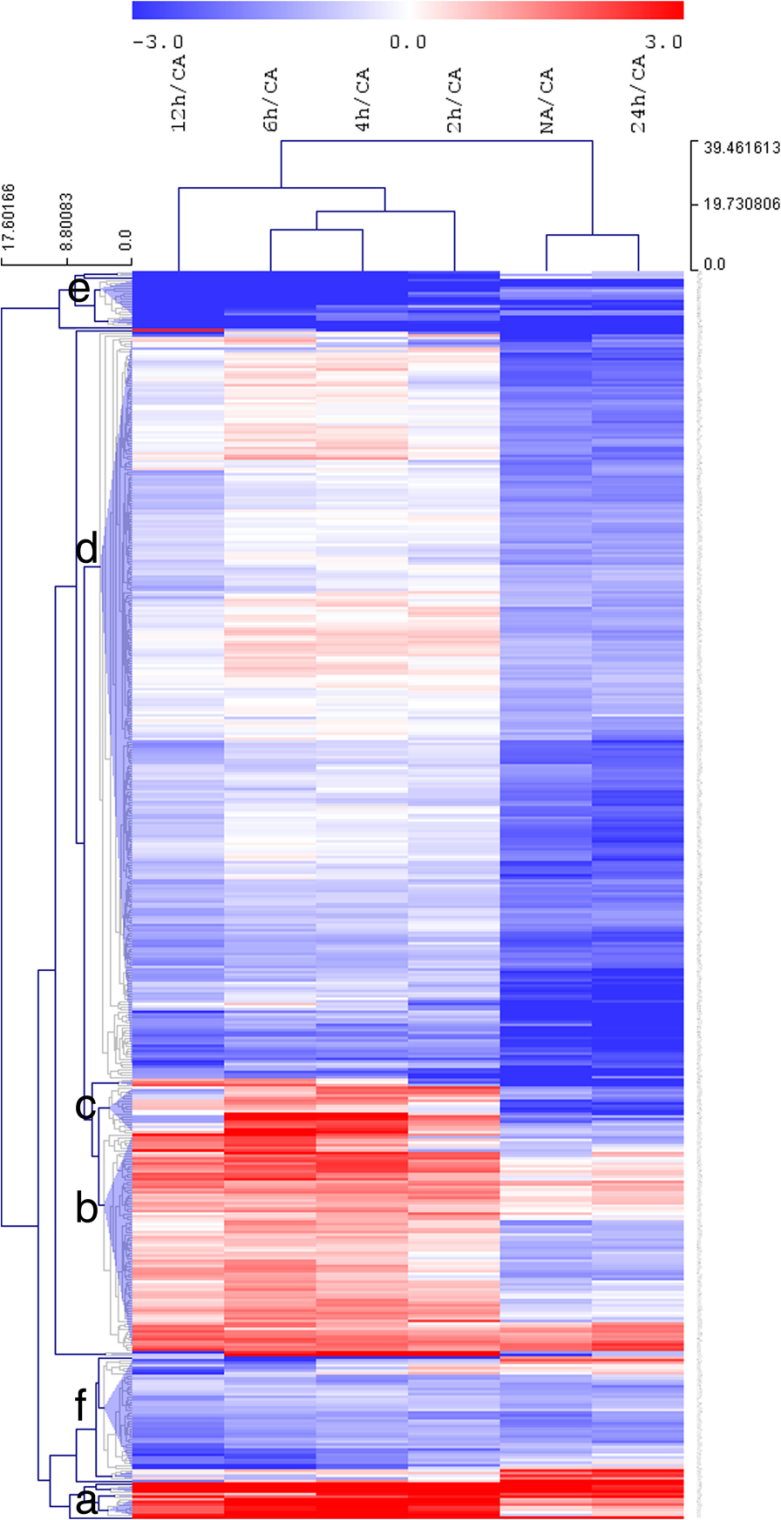
Hierarchical clustering of changes in the expression of genes encoding transcripition factors. Hierarchical trees were drawn, based on log_2_ fold change in relative expression of 476 genes between cold acclimated (CA) and deacclimated and between cold acclimated and non-acclimated (NA) *Arabidopsis* plants. Plants were cold acclimated at 4 °C for three days and thereafter deacclimated at 20 °C for 2 h, 4 h, 6 h, 12 h or 24 h. Genes were selected based on ANOVA analysis to be differentially regulated at least at one time point with a log_2_ fold change of at least + or −1. Lists with the names of all genes in the different clusters indicated by the letters a to f can be found in Additional file 4

To identify transcriptional regulators of the deacclimation process, we determined transcript levels of 1880 TF genes using qRT-PCR [25–27]. After filtering (see Methods for details), expression values of 1462 genes were included in a detailed analysis (Additional file 1). Applying stringent criteria for differential gene expression (FDR *P* < 0.01 and log_2_ fold change of at least −1 or +1) the relative expression of 476 TF genes differed significantly between at least two treatments. In accordance with the PCA, hierarchical cluster analysis (Fig. 2) showed clear differential expression patterns among the treatments, separating the samples into two clusters; one corresponding to 2 h to 12 h of deacclimation and the other encompassing non-acclimated samples and samples deacclimated for 24 h. Clustering further grouped the genes into six major clusters, corresponding to different expression patterns relative to cold acclimated samples and 14 small clusters containing only one or a few genes with distinct expression patterns (Fig. 2 and Additional file 4). The major clusters included (a) seven genes showing an instant, transient increase in expression during deacclimation relative to the acclimated state; (b) 83 genes that also showed a transient increase, although this was less pronounced than in cluster (a); (c) 18 genes that increased transiently during the first 12 h of deacclimation, but were considerably down-regulated at 24 h of deacclimation and (d) 285 genes that were down-regulated at 24 h of deacclimation and to a lesser extent at 12 h of deacclimation. Genes in the last two clusters (e and f) showed a transient decrease, reaching minimum expression levels around 12 h of deacclimation, but their expression relative to cold acclimated samples varied. Genes in cluster (e) were more strongly repressed than genes in cluster (f).

When only considering genes differentially expressed relative to samples from cold acclimated plants, the number of regulated TF genes increased with increasing duration of deacclimation (Table 1). Among those TF genes showing significant differential expression after 2 h of deacclimation, 26 were up-regulated, while 33 were down-regulated compared to their corresponding levels in cold acclimated plants. The number of significantly regulated TF genes after FDR correction was relatively low from 4 to 12 h. After 24 h of deacclimation, the numbers of up- and down-regulated TF genes were similar to those observed in non-acclimated relative to cold acclimated plants, in agreement with the results of the PCA (Fig. 1a).

**Table 1:** The numbers of up- and down-regulated transcription factor (TF) genes and the ratio between up- and down-regulated TF genes following 2 h, 4 h, 6 h, 12 h or 24 h of deacclimation (DEA) at warm temperatures or in non-acclimated (NA) plants

Treatment	Number of genes				Overlap with genes regulated in the opposite direction after 3 d of CA (%)	
	Up-regulated	Down-regulated	Regulated	Ratio	Up-regulated	Down-regulated
2 h DEA	26	33	59	0.79	43	11
4 h DEA	3	12	15	0.25	6	4
6 h DEA	0	5	5	NA	NA	1
12 h DEA	5	16	21	0.31	13	5
24 h DEA	22	284	306	0.08	81	88
NA	16	198	214	0.08	–	–

Numbers indicate the genes out of the 476 shown in Fig. 2 that were significantly (FDR *P* < 0.05) regulated relative to cold acclimated (CA) plants

To identify TF gene families whose members were predominantly regulated during deacclimation, we performed an overrepresentation analysis for the 24 h time point and for the data from non-acclimated plants. Among up-regulated TF genes, the basic helix-loop-helix (bHLH) family was overrepresented at both time points, while the heat shock factor (HSF) family was overrepresented only in non-acclimated plants (data not shown). The number of overrepresented TF families among down-regulated genes was much higher, in agreement with the larger number of significantly down-regulated genes (Table 2). Most families overrepresented after 24 h of deacclimation were also overrepresented among TFs with a reduced expression in non-acclimated relative to cold acclimated plants. Since the TF genes identified as down-regulated in non-acclimated plants in this analysis are up-regulated in the reverse comparison (i.e. they are cold induced), this indicates that TF genes induced during cold acclimation are rapidly down-regulated during deacclimation.

**Table 2:** Transcription factor families significantly enriched in down-regulated genes following 24 h of deacclimation (DEA) and in non-acclimated (NA) relative to cold acclimated plants

bin	Transcription factor family	24 h DEA	NA
27.3.11	C2H2 zinc finger family	X	X
27.3.7	C2C2(Zn) CO-like, Constans-like zinc finger family	X	X
27.3.26	MYB-related transcription factor family	X	X
27.3.3	AP2/EREBP, APETALA2/Ethylene-responsive element binding protein family	X	X
27.3.8	C2C2(Zn) DOF zinc finger family	X	
27.3.32	WRKY domain transcription factor family	X	X
27.3.35	bZIP transcription factor family	X	
27.3.25	MYB domain transcription factor family	X	X
27.3.22	HB,Homeobox transcription factor family	X	
27.3.62	Nucleosome/chromatin assembly factor group	X	X
27.3.16	CCAAT box binding factor family, HAP5	X	X
27.3.20	G2-like transcription factor family, GARP	X	X
27.3.9	C2C2(Zn) GATA transcription factor family	X	X
27.3.15	CCAAT box binding factor family, HAP3	X	X
27.3.18	E2F/DP transcription factor family	X	
27.3.12	C3H zinc finger family		X
27.3.13	CCAAT box binding factor family, DR1	X	
27.3.2	Alfin-like	X	X
27.3.30	Trihelix, Triple-Helix transcription factor family	X	X
27.3.4	ARF, Auxin Response Factor family	X	X
27.3.49	GeBP like	X	X
27.3.57	JUMONJI family	X	
27.3.6	bHLH,Basic Helix-Loop-Helix family	X	X
27.3.63	PHD finger transcription factor	X	X
27.3.24	MADS box transcription factor family		X
33.3	development.squamosa promoter binding like (SPL)	X	

Fields with an X indicate significant enrichment of specific families

### Global changes in gene expression during deacclimation

To investigate whether deacclimation is simply a reversal of cold acclimation or whether loss of acclimated freezing tolerance and the return to an unstressed phenotype involves additional changes in gene expression, a global transcriptomic study was carried out using microarrays. 1462 TF genes investigated by qRT-PCR were also represented on the microarrays and were used to validate the transcriptome data. Data from the two platforms showed a high correspondence (Additional file 5), with only a small number of genes showing strong regulation in one dataset, but no change in the other.

The number of regulated genes increased with increasing duration of deacclimation (Table 3). After 24 h the expression of 2335 genes was significantly affected by the deacclimation treatment. Different durations of deacclimation affected transcript levels in different ways. Among those genes showing differential expression after 2 h, 811 were up- while 495 were down-regulated, compared to acclimated plants. Over time the number of regulated genes increased, in particular for repressed genes. Thus, the ratio of induced to repressed genes (Table 3) indicated more up- than down-regulated genes during the early phase of deacclimation, while after 12 h and 24 h down-regulation predominated. A total of 230 and 128 genes were commonly up- or down-regulated at all time points. According to an overrepresentation analysis the functional classes heat stress (bin 20.2.1) and brassinosteroid synthesis and degradation (bin 17.3.1.2.99) were significantly enriched in commonly up-regulated genes, while genes associated with ribosome biogenesis and pre-rRNA processing and modifications (bin 29.2.2.3.4) were overrepresented among the consistently down-regulated genes.

**Table 3:** The numbers of up- and down-regulated genes and the ratio between up- and down-regulated genes following 2 h, 4 h, 6 h, 12 h or 24 h of deacclimation (DEA) at 20 °C of cold acclimated plants of *Arabidopsis thaliana*

Treatment	Up-regulated	Down-regulated	Regulated	Ratio (up/down)	Overlap with genes regulated in the opposite direction after 3 d of CA (%)	
					Up-regulated	Down-regulated
2 h DEA	811	495	1306	1.64	34	23
4 h DEA	902	766	1668	1.18	36	31
6 h DEA	1155	1025	2180	1.13	38	34
12 h DEA	1197	1381	2578	0.87	46	52
24 h DEA	1138	1197	2335	0.95	72	71
NA	1135	1182	2317	0.96	–	–

Also shown are the overlaps between genes differentially expressed in opposite directions after 3 d of cold acclimation (CA) and following different durations of deacclimation

There were 1135 up- and 1182 down-regulated genes after 3 d of cold acclimation in comparison to non-acclimated plants. Of the genes that were significantly up-regulated during cold acclimation, 23% were down-regulated during the first 2 h of deacclimation (Table 3). Among genes down-regulated during cold acclimation, 34% were significantly induced after 2 h of deacclimation. After 24 h of deacclimation, the overlap with genes differentially expressed in the opposite direction during cold acclimation had increased to 72%.

### Functional analysis of genes responsive to deacclimation

**Fig. 3:**
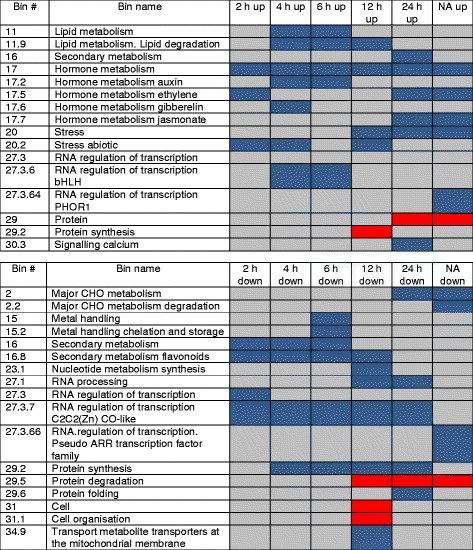
Overrepresentation analysis of genes significantly changed in expression during deacclimation and in non-acclimated (NA) relative to cold acclimated plants. Genes were grouped in MapMan bins and overrepresentation was determined for genes showing either significant up-regulation (top) or down-regulation (bottom) of expression during deacclimation. Blue color indicates significant enrichment of up- or down-regulated genes, red indicates significant depletion. Except for the bin RNA regulation of transcription only the two highest MapMan bin levels are shown. Significant bins at all levels can be found in Additional file 6

Overrepresentation analysis was used to identify functional classes that contained a significantly higher or lower number of genes with significantly changed expression than could be expected by chance (Fig. 3). A complete overview of the respective bins and sub-bins is given in Additional file 6. This analysis revealed physiological processes that are predominantly regulated at the level of gene expression when cold acclimated plants are subjected to warm temperatures. Among up-regulated genes hormone metabolism was overrepresented at all time points, including processes related to auxin, ethylene, gibberellin, jasmonate and brassinosteroid metabolism. The overrepresentation of brassinosteroid metabolism was only apparent at lower bin level (Additional file 6). Genes associated with lipid degradation and abiotic stress were over-represented among genes up-regulated at early time points up to 12 h of deacclimation. The enrichment of the abiotic stress bin was predominantly due to an overrepresentation of heat stress related genes, while the subgroups of the lipid metabolism bin that were overrepresented among up-regulated genes were mainly those involved in fatty acid synthesis and elongation, synthesis of steroids/squalene, and degradation by lipases, lysophospholipases and beta-oxidation.

Among down-regulated genes, the functional classes of secondary metabolism and protein synthesis showed overrepresentation at 2 h to 12 h and 4 h to 24 h of deacclimation, respectively. The corresponding secondary metabolism genes were associated with glucosinolate and flavonoid metabolism, while the protein synthesis genes were all connected to ribosome biogenesis. Genes associated with nucleotide metabolism, major carbohydrate (CHO) metabolism, protein folding and RNA processing were overrepresented during the latest time points, indicating that these processes were only slowly down-regulated during deacclimation. Interestingly, protein degradation was under-represented among down-regulated genes after 12 h and 24 h of deacclimation.

To a large extent the same functional classes were significantly enriched in up-regulated genes in non-acclimated plants and in plants deacclimated for 24 h. However, they differed with respect to functional classes with an overrepresentation of down-regulated genes, in particular protein synthesis and sub-bins of the class major CHO metabolism, indicating that these processes were either not fully reversed within 24 h or that they may be specific to deacclimation.

### Primary metabolism during deacclimation

**Fig. 4:**
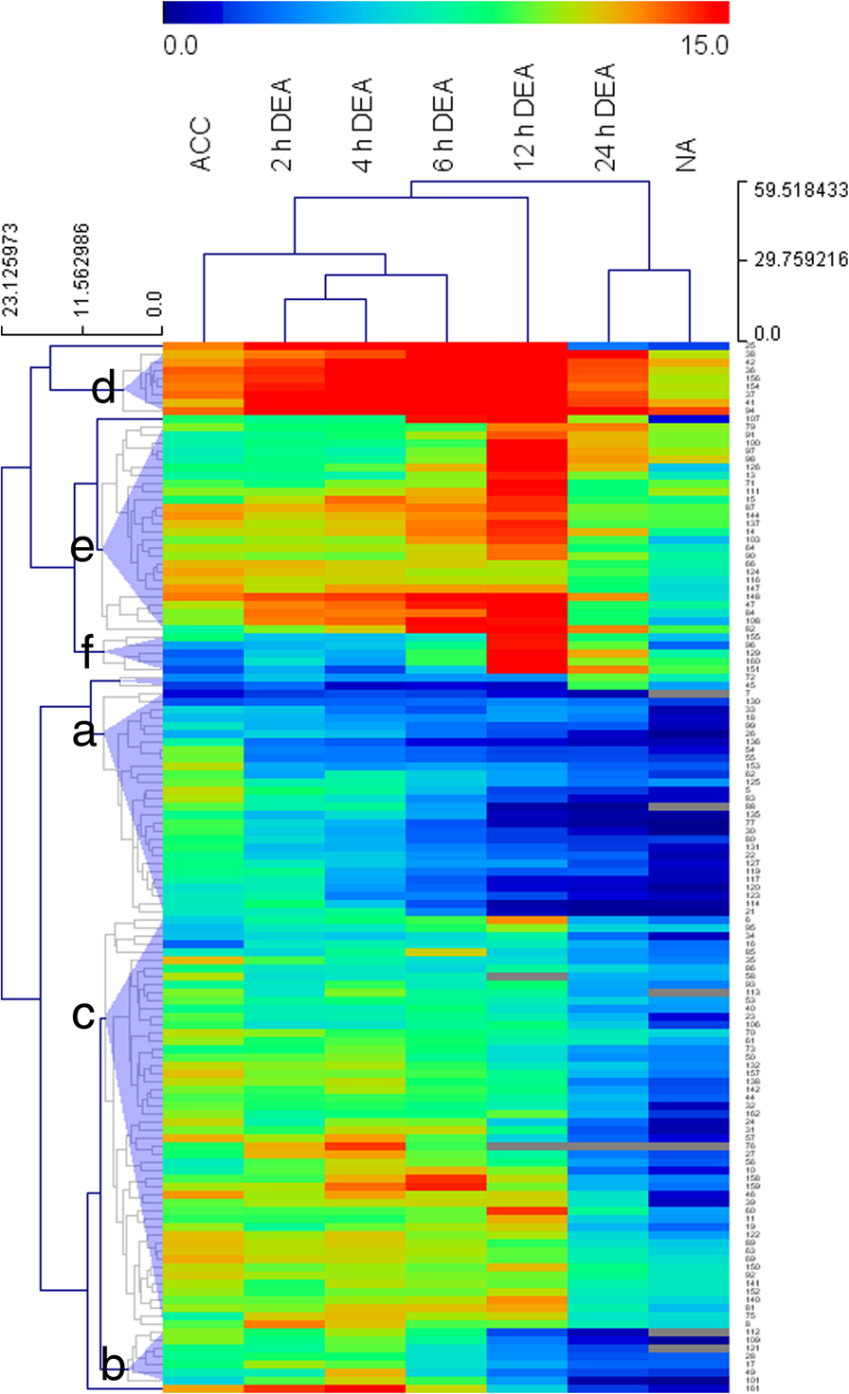
Global overview of the dynamic changes in primary metabolite pool sizes during deacclimation of *Arabidopsis thaliana*. Plants were non-acclimated (NA) or cold acclimated at 4 °C for 3 d (ACC). Cold acclimated plants were then deacclimated (DEA) at 20 °C for 2 h, 4 h, 6 h, 12 h and 24 h. The metabolic profiles were statistically analyzed using a one-way ANOVA at FDR *P* < 0.05 by comparing all conditions against each other. Metabolites that showed significant changes between treatments were clustered using Euclidian distance and average linkage. Lists with the names of all metabolites in the different clusters indicated by the letters a to f can be found in Additional file 7. The numbers on the right side of the heat map refer to the numbering of the metabolites in Additional file 3

The transcriptomic analysis was complemented with an analysis of primary metabolites, such as sugars, amino acids and organic acids as these have been extensively studied in relation to cold acclimation [2, 3]. A total of 130 metabolites showed significantly changed pool sizes during deacclimation or between cold acclimated and non-acclimated plants. Hierarchical clustering (Fig. 4) revealed, in accordance with the PCA (Fig. 1c), that samples from plants after 2 h and 4 h of deacclimation clustered closest together. Further, samples from cold acclimated plants and also from plants deacclimated for up to 6 h were separated from non-acclimated plants and from plants deacclimated for 12 h and 24 h.

In addition, the clustering revealed six major patterns of metabolic responses (Fig. 4, Additional file 7): (a) Metabolites that showed an early decrease in abundance during deacclimation, including six amino acids, and sugars and sugar-phosphates implicated in cold acclimation (Glc, Fru, Raf, Glc-6-P, Fru-6-P); (b) metabolites with an intermediate decrease pattern with the highest amounts in cold acclimated plants and during the early phase of deacclimation (2 h – 4 h) and gradually decreasing amounts between 6 h and 24 h; (c) metabolites with little variation in pool size between cold acclimated plants and plants deacclimated for 2 h to 12 h, but strongly reduced content after 24 h and in non-acclimated plants. This was the largest cluster, containing 51 compounds dominated by acids, amino acids, unknown compounds, N-compounds, phosphates and sugars. The other three clusters (d, e, f) showed a transient increase in metabolite abundance after 12 h of deacclimation. (d) Metabolites present in relatively high amounts under all conditions. This group included three fatty acids, two N-compounds and three unknown compounds. (e) Metabolites highly abundant at intermediate time points. These included primarily unknown compounds and cell wall-related sugars such as arabinose, psicose, xylose and galactose. (f) Metabolites found in relatively low amounts in all treatments except after 12 h of deacclimation (five unknown compounds). In addition, four small clusters were identified, containing one to three metabolites with distinct accumulation patterns, including glycine, panthenol and glycerol.

### Influence of diurnal regulation on TF gene expression and metabolite abundance during deacclimation

In *Arabidopsis*, cold (4 °C) has massive effects on the expression of diurnally regulated genes, which partly extends to the regulation of primary metabolism [25, 38]. Under diurnal conditions cold reduces the amplitude of cycles of clock components and dampens or disrupts the cycles of output genes. It is unknown, however, how fast clock oscillator components and output genes increase their expression amplitudes upon transfer to warm conditions. Hence, we considered overlap of TFs and metabolites known to be diurnally regulated with TFs and metabolites transiently regulated during deacclimation.

Of the 476 TF genes whose relative expression differed significantly between at least two treatments, 204 were merely differentially expressed between cold-acclimated and non-acclimated plants and/or plants deacclimated for 24 h, indicating no diurnal oscillations. Using manual inspection and pair-wise t-tests between cold acclimated samples and samples deacclimated for up to 24 h, the remaining 272 differentially expressed TF genes were divided into two groups; one of 186 genes that predominantly decreased or increased in expression or showed no clear change over time and one of 86 genes that were transiently up- or down-regulated. For 49 of these genes, regulation was significant at *P* < 0.05 without FDR correction, while 37 genes remained with the more stringent FDR corrected *P* < 0.05 (Additional file 8). In the hierarchical cluster analysis (Fig. 2) 64 of these 86 genes belonged to clusters (a), (b) and (c), which included transiently increasing genes. Hence, all genes in cluster (a), 12 of the 18 genes in cluster (c) and 45 of the 83 genes in cluster (b) were transiently regulated during deacclimation. Twelve genes belonged to clusters (e) and (f), showing a transient decrease. The remaining ten genes belonged to small clusters containing only a few genes with distinct expression patterns. Interestingly, *CCA1* and *LHY1*, encoding central components of the circadian clock were included in these small clusters. Thirty-seven of the transiently regulated TF genes (without FDR correction) are diurnally regulated under warm, long-day (16 h) ([38]; 31 genes) and/or warm, short-day (12 h) ([39]; 6 additional genes) conditions, while another 35 transiently regulated TF genes did not cycle under warm conditions [38, 39]. These genes are labeled in Additional file 8.

Thirty-four metabolites were identified as transiently regulated in the same way as transiently regulated TF genes (Additional file 9). In the cluster analysis, these metabolites were contained in clusters (d), (e), (f) and, to a lesser extent, (c). Clusters (d), (e) and (f) were all characterized by metabolites showing a transient increase in pool size after 12 h of deacclimation. Only two of these metabolites showed diurnal oscillations under warm conditions [38]. In contrast, 15 metabolites previously shown to be diurnally regulated generally decreased in abundance during 24 h of deacclimation, while three metabolites, which did not exhibit diurnal oscillations under warm, long-day conditions, transiently increased in pool size during deacclimation.

## Discussion

### Deacclimation is in part a reversal of cold acclimation

Collectively, our data indicate a temporally regulated response of cold acclimated *Arabidopsis* plants to warm temperatures. With respect to the temporal coordination of responses at different molecular levels, transcriptional responses underlying deacclimation appear faster than metabolic responses. Among transcriptional responses, the global composition of the transcriptome approximated that of non-acclimated plants after 24 h of deacclimation, while for the TF genes transcript composition was virtually identical at these time points.

Transfer of cold acclimated *Arabidopsis* to warm conditions lead to massive changes in global transcript levels, suggesting that ca. 8% of the *Arabidopsis* genome is differentially expressed during deacclimation. The numbers of significantly regulated genes following 12 h and 24 h of deacclimation were close to the 2588 regulated genes detected over time during cold acclimation [23], but a different microarray and no fold-change cut-off was applied in the earlier study, making a direct comparison difficult. The majority of genes significantly induced or repressed during deacclimation overlapped with genes differentially expressed in the opposite direction when comparing transcript levels in non-acclimated and cold acclimated plants, in agreement with previous reports [14, 20]. Reversal of cold acclimation during deacclimation is also apparent at the level of global functional analysis. Functional groups with a significant overrepresentation among up-regulated genes during deacclimation and among down-regulated genes during cold acclimation included stress regulated genes and hormone metabolism, while major CHO metabolism behaved in the opposite way.

All three cold induced *CBF* genes showed a decrease in expression within the first two hours of deacclimation followed by a transient increase, reaching expression levels comparable to the expression in non-acclimated plants after 24 h. Despite similar expression patterns over time, *CBF2* showed greater changes in expression than *CBF1* and *CBF3* and the effect of deacclimation duration on expression in the ANOVA analysis was only significant for *CBF2.* This may suggest that CBF2 has a different function in deacclimation than CBF1 and CBF3 in agreement with a different regulation of *CBF*2 compared to *CBF1* and *CBF3* during cold acclimation [40] and the recent suggestion from mutational studies that CBF2 is more important for acclimated freezing tolerance than CBF1 and CBF3 [41].

### Metabolic regulation during deacclimation

Similar to cold acclimation, deacclimation is also associated with many metabolic changes. Decreasing pool sizes of Glc, Fru and Raf early during deacclimation are in agreement with biochemical measurements of these solutes and sucrose after 24 h of deacclimation [17]. These three sugars together with galactinol and the unknown compound A196004 were also identified as important predictors of freezing tolerance in *Arabidopsis* [42] suggesting that their rapid decrease may be functionally related to the rapid loss of freezing tolerance observed after one day of deacclimation [17]. Through mass spectrometric analysis A196004 was tentatively identified as a small hexose conjugate [42]. Further work will be necessary for a definitive identification of this compound.

Our data suggest that sugars accumulated during cold acclimation were used as energy and carbon sources during deacclimation. Concomitant with reduced sugar content in deacclimating plants, the functional class of major CHO metabolism was significantly enriched in down-regulated genes, mostly encoding enzymes for starch and sucrose metabolism. In the bin sucrose degradation, up-regulation of the vacuolar invertase gene *βfruct3* may suggest sucrose hydrolysis to fuel cell expansion due to resuming growth [43]. In addition, the pool sizes of all identified glycolytic intermediates except pyruvate (Glc, Glc-6-P, Fru-6-P, PEP) decreased during deacclimation, supporting the interpretation that carbon is withdrawn from these pools during deacclimation and thus indicating a link between changes in di- and trisaccharides and glycolytic intermediates.

Most TCA cycle intermediates and amino acids accumulate in cold acclimating *Arabidopsis* [42, 44] and showed a decrease during deacclimation. The functional significance of a decrease in TCA cycle intermediates during deacclimation is unclear, but concomitantly decreasing pool sizes of glycolytic intermediates suggest a depletion of both TCA cycle and glycolytic intermediates that is likely due to increased respiratory energy production. This interpretation is in agreement with a transient up-regulation of the *HRA1* and *HRE2* TF genes that control hypoxia tolerance in plants [45]. *HRA1* functions in attenuating hypoxia responses in young tissues and meristematic regions by dampening low-oxygen responses under aerobic conditions in regions of the plant that are experiencing physiological hypoxia. Hence, transient up-regulation of these genes together with the described metabolic signature may be signs of high respiratory activity associated with growth resumption under deacclimating conditions. Oono et al. [20] also identified *HRA1* as up-regulated after 1 h and in particular 5 h of deacclimation. Resuming growth requires activation of protein biosynthesis and will cause enhanced amino acid consumption. Amino acid consumption may further enhance carbon depletion from glycolysis and TCA cycle intermediates for the biosynthesis of the pyruvate, the aspartate and the glutamate families of amino acids.

Interestingly, the overrepresentation of protein synthesis-related genes among down-regulated transcripts was confined to genes associated with ribosome biogenesis, indicating that the cells economize on amino acids by preferentially using ribosomes already present in the cells, at least for the initial phase (24 h) of deacclimation. In *Hydrangea paniculata*, deacclimation is characterized by a distinct decrease in the abundance of predominantly stress- and defence-related proteins [46] and it can be assumed that the degradation of freezing tolerance-related proteins and a remobilization of the resulting amino acids for protein biosynthesis also takes place in *Arabidopsis*. Proline, arginine, glutamine and γ-amino butyric acid (GABA), which showed a late decrease during deacclimation, are members of the glutamate family of amino acids originating from α-ketoglutarate. Proline is a well-known cryoprotectant with compatible solute properties and consistent with our data, biochemical measurements showed strongly reduced pool size within 24 h of deacclimation [17]. The decrease in GABA content suggests that its potential role in signaling under stress conditions [47] is alleviated during deacclimation. This is in line with observations in wheat, where the content of GABA decreased significantly in cold acclimated plants recovering from freezing at −3 °C [48]. In cold acclimating *Arabidopsis* accumulation of branched-chain amino acids is thought to be part of a preemptive defense response against opportunistic attack by pathogens [44]. Transfer to warm conditions may diminish the need for such a defense response, as indicated by decreases in isoleucine and valine content during deacclimation. Similarly, decreasing contents of the aspartate-derived amino acids homoserine and threonine, which may provide increased plant pathogen resistance [49], may also reflect a reduced need for pathogen resistance.

Many of the changes in primary metabolite content during deacclimation were not globally reflected at the transcript level. Hence, the functional classes glycolysis, TCA/organic transformation and amino acid metabolism were not overrepresented among regulated genes, and although there were changes in the pool sizes of many carbohydrates already after a few hours of deacclimation, the functional group of major CHO metabolism was only overrepresented among down-regulated genes after 24 h. This implies that at least for some pathways decreases in metabolite levels are independent of transcript abundance. In cold acclimating plants post-transcriptional and post-translational regulation are key parts of metabolic adjustment [2]. The present study suggests that both levels of regulation play important roles also during deacclimation. In addition it should be noted that metabolite levels will not be directly influenced by reduced transcript abundance, but rather by a complex balance between the stability of enzymes that lead to metabolite accumulation and the activation/de-novo synthesis of degradation enzymes. Unfortunately, nothing is known about how a change in temperature as applied here for deacclimation affects the stability of the enzymes that are responsible for the observed changes in metabolite pool sizes.

Up-regulation of lipid metabolism may be related to increased carbon demand due to growth resumption and suggests remobilization of storage lipids that accumulate in the cold [50]. This could be traced at the metabolite level, where the contents of the three fatty acids palmitic, stearic and myristic acid increased transiently during deacclimation. Although not visible at the transcriptional level, modification of cell wall properties may also be involved in deacclimation, as the contents of five monosaccharides required for cell wall biosynthesis increased transiently during deacclimation (galactose, xylose, arabinose) or showed a late decrease (mannose, fucose).

Repression of transcripts associated with flavonoid metabolism during the first 12 h of deacclimation is in agreement with previous evidence for a role of flavonoid metabolism in *Arabidopsis* freezing tolerance [51–54]. The functional role of secondary metabolites in plant freezing tolerance, however, is not clear.

### Heat stress responses during deacclimation

The heat stress bin was overrepresented among up-regulated genes after 2 h, 4 h and 12 h of deacclimation. Nine members of the HSF gene family were significantly regulated during deacclimation. HSFs play the major role in activating the transcription of heat-induced genes [55]. Although 20 °C is not normally a heat stress for *Arabidopsis*, the sudden up-shift in temperature by 16 °C was apparently perceived as heat stress by the plants. In addition, at least HSFA1 proteins play a role in tolerance to mild temperature increases, well below those associated with heat stress responses [56]. Knowledge about the specific roles of different HSFs is limited, but HSFA1E, which was repressed during deacclimation, is likely involved in osmotic stress tolerance and does not confer thermotolerance [56]. HSFA1A, HSFA1B and HSFA2 are activators of transcription crucial for thermotolerance in plants, while HSFB1 is a transcriptional repressor, negatively regulating the expression of other HSFs [56, 57]. HSFA3, which was up-regulated during deacclimation, mediates signalling by DREB2A, a dehydration- and cold-responsive TF [57]. Accordingly, *DREB2A* was significantly down-regulated after 24 h of deacclimation.

### The involvement of hormone metabolism in deacclimation

Hormone metabolism was the only functional group overrepresented among up-regulated genes at all time points, indicating the relevance of altered hormonal regulation during deacclimation. Significant enrichment of genes related to the metabolism of auxin, gibberellin (GA) and brassinosteroids (BR) among up-regulated genes is likely related to growth resumption in response to warm temperatures, while cold results in an overrepresentation of auxin-induced and BR-responsive genes among down-regulated genes [23, 51] and a reduced level of bioactive GA [58].

Several lines of evidence point to an important role of auxin in deacclimation. The Aux/IAA genes are key regulators of auxin-modulated gene expression that are themselves auxin inducible [59]. The expression of *IAA5*, *IAA19* and *IAA29* increased transiently during deacclimation. A major hub in the ambient temperature signaling network is the basic helix-loop-helix (bHLH) TF PIF4. At high temperature, the PIF4 protein binds to the promoters of auxin biosynthesis and response genes promoting auxin biosynthesis and growth [60]. In accordance with a role of PIF4 in acclimation to high temperature a moderate increase in growth temperature results in a fast and transient peak in *PIF4* expression [61]. During deacclimation, *PIF4* showed a similar expression pattern, suggesting that PIF4 may also be involved in the regulation of auxin metabolism under these conditions. *PIF4* has previously been reported to be up-regulated following 1 h and 5 h of deacclimation [20]. *IAA19* and *IAA29*, which were among the transiently up-regulated Aux/IAA genes, are both directly activated by PIF4 [62]. Also, the *SAUR* genes 19, 20, 21, 22, 23 and 24 were significantly up-regulated at 4 h and 6 h of deacclimation. *SAUR19–24* are likely all PIF4 target genes which promote hypocotyl elongation [63]. Despite the indications of PIF4 function in auxin mediated growth resumption upon transfer of cold acclimated plants to warm temperatures, our gene expression analysis only provides limited evidence that auxin concentrations are controlled at the transcriptional level. The only relevant changes in gene expression were an up-regulation of *YDR1/GH3.2*, *GH3.3* and *ILL6* after 4 h and/or 6 h of deacclimation. YDR1/GH3.2 and GH3.3 belong to the same clade of group II GH3 proteins that conjugate IAA to amino acids in vitro and are therefore predicted to decrease endogenous IAA levels, while ILL6 can release IAA from conjugates [64].

Four genes encoding GA biosynthesis enzymes were significantly up-regulated during deacclimation. GA20ox and GA3ox control oxidation steps in the production of growth-active GAs [65], suggesting that the return to an unstressed phenotype involves an increase in bioactive GAs. GAs stimulate growth by activating the degradation of DELLA proteins which repress growth. The *Arabidopsis* genome harbours five DELLA genes: *GAI*, *RGA*, *RGL1*, *RGL2* and *RGL3* [66] and *GAI*, *RGL1 and RGL2* were significantly regulated during deacclimation. Up-regulation of *GAI* during deacclimation has been reported previously [20]. GAI and RGL1 and 2 are involved in controlling cell expansion, cell division and floral development [66], indicating tight developmental control in deacclimating plants. GA metabolism is also linked to PIF4-dependent elongation growth. Hence, DELLA proteins restrain PIF4-dependent growth by sequestering PIF4. GA-mediated proteasomal degradation of DELLA proteins releases this constraint on PIF4, thereby stimulating growth [61].

The significant overrepresentation of BR metabolism among up-regulated genes was due to six genes encoding enzymes of the BR biosynthetic pathway that were transiently induced, including *DET2*, *BAS1*, *SQE2*, *SQE5* and *SQE6*. *SQE2*, *SQE5* and *SQE6* are down-regulated during cold acclimation [23]. *DET2* encodes a reductase in the brassinolide biosynthetic pathway, while BAS1 is a brassinosteroid-inactivating enzyme [67].

JA regulates a wide spectrum of plant processes, such as growth and development, as well as stress defence. Both the JA-related TFs *JAM1* and *JAS1* and the biosynthesis genes *LOX1*, *AOC* and *OPR3* [68] were significantly up-regulated in plants during deacclimation. This indicates that deacclimation is associated with increased endogenous JA levels and altered response regulation as *JAM1* is a negative regulator of JA responses [69] and *JAZ10* encodes a JASMONATE ZIM-DOMAIN (JAZ) protein. JAZ proteins act as transcriptional repressors of JA-responsive genes [68].

ACC synthase and ACC oxidase are the rate-limiting enzymes in ethylene biosynthesis and transcriptional regulation of *ACS* and *ACO* genes is a pivotal mechanism controlling this process [70]. Up-regulation of *ACO1* and two or three *ACS* genes after 2 h (*ACS8*, *ACS11*) and 24 h (*ACS1*, *ACS6*, *ASC11*) of deacclimation indicate that ethylene may have a significant role in controlling responses to increased temperature.

### Regulation of morphogenesis during deacclimation

It should be mentioned that, while we saw clear evidence for a transition to growth and development at the transcript and metabolite levels, there were no phenotypic changes visible in the plants during this short deacclimation period of 24 h. However, several families of TF genes that were up-regulated during deacclimation have members controlling fundamental aspects of plant development, such as the CONSTANS (CO)-like and the Squamosa promoter binding-like families whose members control photoperiodic regulation of flowering and are also involved in branching and determining leaf initiation rate [71]. Similarly, members of the zf-HD, HB and AS2 Lateral Organ Boundaries families are predominantly associated with plant developmental regulation such as defining organ boundaries and floral development [71–73], including *BBX27* and *LBD41* which were transiently up-regulated during deacclimation and are involved in controlling growth and other developmental processes [71, 74]. The bHLH family was significantly overrepresented among up-regulated TF genes after 24 h of deacclimation. The bHLH members so far characterized, including *PIF4* discussed above, function in regulation of fruit dehiscence, carpel, anther and epidermal cell development, phytochrome signaling, flavonoid biosynthesis, hormone signaling and stress responses [75]. These findings emphasize that loss of freezing tolerance and growth resumption are interrelated processes that are both transcriptionally highly regulated.

Many genes in clusters (a), (b) and (c) of the hierarchical cluster analysis of expression changes of significantly regulated TF genes are of particular interest as regulators of deacclimation because they are transiently up-regulated. Except for *PIF4* whose transcription is controlled by the circadian clock [61] and *BBX27*, which is diurnally regulated under warm, short-day conditions [39], the genes in cluster (a) have not been described as diurnally regulated, indicating that their transient accumulation pattern was indeed a deacclimation response. However, even for *PIF4* we observed a fast transient increase in expression in the morning upon transfer to warm conditions. This expression pattern does not fit the diurnal expression pattern of *PIF4*, which is lowest at midnight, increases towards dawn and peaks at midday [61], indicating that also *PIF4* expression is strongly influenced by deacclimation. Other TF genes transiently induced during deacclimation but not regulated by the clock include *PIL1–2* that belongs to the same small PIF/PIL subfamily of bHLH TFs as *PIF4*. PIFs/PILs have diverse functions in light-mediated plant development, with PIL1 having photomorphogenesis-related functions [76]. Members of the bHLH family in this cluster also included *FBH1*, *FBH3* and *HEC1*, which are all regulators of growth and developmental processes. FBH1 and FBH3 positively regulate *CONSTANS* transcription for photoperiodic flowering [77]. Interestingly, FBH1 is a transcriptional modulator of ambient temperature signaling and clock responses in *Arabidopsis* by regulating *CCA1* expression [78]. HEC1 contributes to shoot apical meristem function by promoting stem cell proliferation [79]. Clusters (b) and (c) also included several members of other TF families linked to growth and development, such as *ATH1* [80], *WLIM2A* [81], *TCP7* [82], *NF-YB3* and *NF-YC4* [83].

### Regulation of the circadian clock during deacclimation

Since we found evidence for transiently regulated genes and an, at least partial, reactivation of the circadian clock in our expression data, we analyzed the expression patterns of clock components in more detail. Especially clusters (b), (e), (f) and one of the small clusters with only two members included several TF genes that are part of the circadian clock, including the central oscillator components *CCA1* and *LHY*. Both genes are morning expressed under ambient temperature conditions [84] and their expression also peaked in the morning and decreased during the day during deacclimation. The log_2_ relative expression amplitudes of *CCA1* and *LHY* were similar to the high amplitudes previously determined under warm, long-day conditions [25]. Further examples of transiently regulated clock components are *NOX* in cluster (c), *EPR1/RVE7* in cluster (b) and *RVE1* in cluster (e). *NOX* is a circadian clock component [84] and *EPR1*/*RVE7* is a component of a slave oscillator involved in the fine-tuning of the circadian rhythm [85]. *RVE1* is homologous to *CCA1* and *LHY*, and plays a role in controlling auxin levels [86]. It is, however, also a negative regulator of acclimated freezing tolerance [87] and its transient up-regulation may therefore also have a direct function in regulating the loss of freezing tolerance during deacclimation. In addition, although not on the list of diurnally regulated genes, *RVE8* was found in cluster (f), which includes transiently down-regulated TF genes. *RVE8* is a morning-phased clock component inducing evening-expressed clock genes [84]. The finding of several clock components among the transiently regulated TFs supports the hypothesis that at least some clock components are rapidly reactivated after transfer from 4 °C to warm conditions, resulting in diurnal regulation of down-stream genes within hours.

Contrary to expectation, primary metabolism appeared to be dominated by a time-dependent process of resetting the acclimation response to the non-acclimated state. Most of the identified metabolites known to be diurnally regulated [38] constantly decreased in abundance during deacclimation, suggesting that temperature responses of primary metabolism dominated over diurnal regulation during the first 24 h. Alternatively, we might have been unable to identify beginning diurnal oscillations in metabolite pools, as we only sampled material during the day and not during the dark period. Only two of the compounds that showed transient changes in abundance during deacclimation are diurnally regulated [38], indicating that the transient accumulation of the remaining metabolites was in fact a deacclimation response.

## Conclusions

**Fig. 5:**
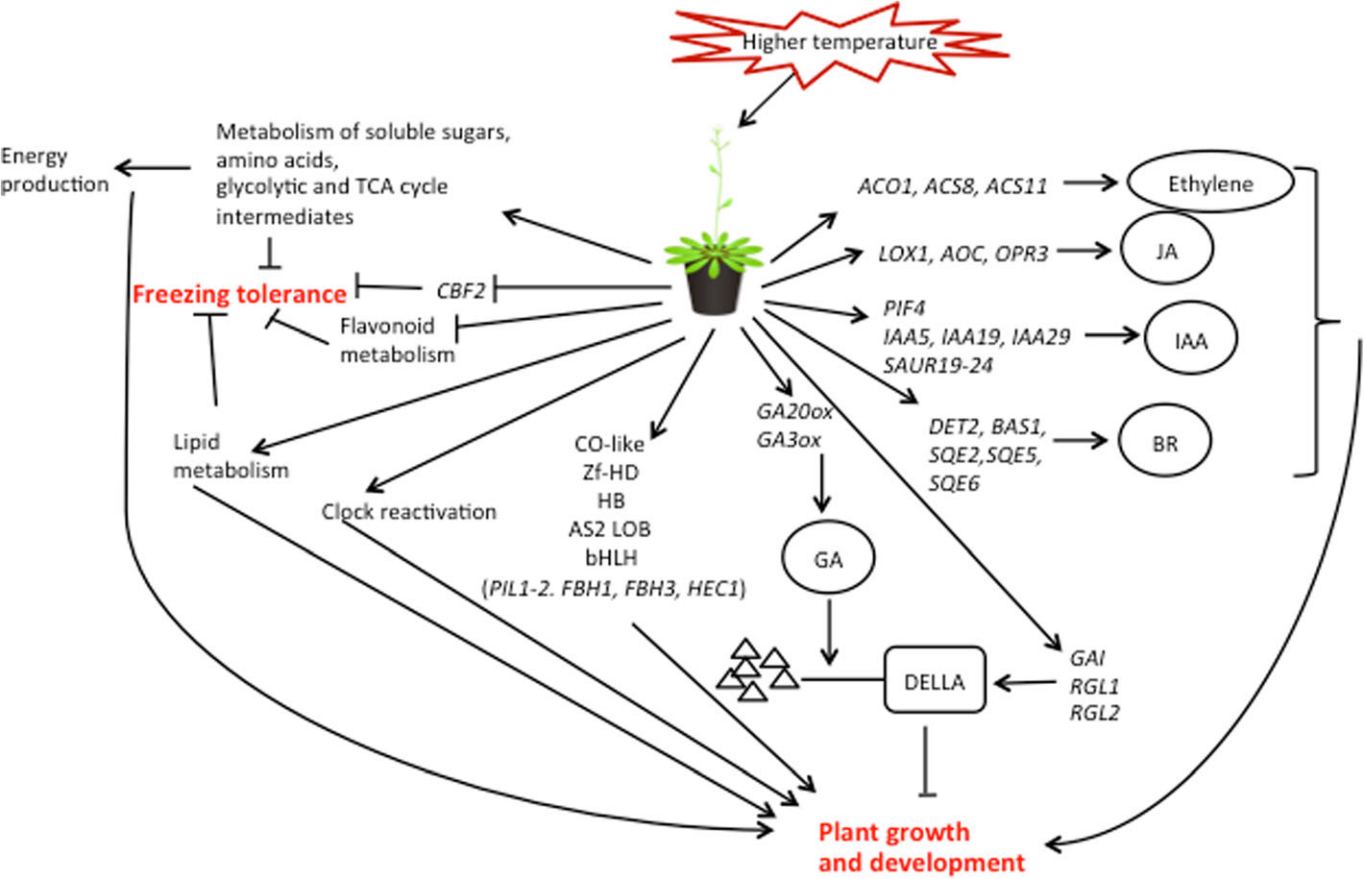
Model summarizing early metabolic and transcriptomic responses of *Arabidopsis thaliana* to deacclimating conditions. Arrows indicate activation or positive influence, lines ending in a T indicate inactivation or negative influence. The two main physiological outcomes (freezing tolerance; growth and development) are highlighted

During deacclimation plants rapidly lose the freezing tolerance they had acquired during cold acclimation. This study provides an analysis of the transcriptomic and metabolomic regulation during the first 24 h of this process. The data indicate two inter-related processes that result in a reduction of freezing tolerance and a reinitiation of growth and development (Fig. 5). Warm temperatures increase biosynthesis and/or bioactive levels of several growth stimulating hormones and induce TFs regulating morphogenesis. Growth resumption is likely fueled by catabolism and interconversion of sugars, amino acids and storage lipids accumulated during cold acclimation. Decreasing levels of sugars and amino acids and down regulation of flavonoid metabolism may cause loss of freezing tolerance. However, warm temperatures also suppress freezing tolerance through down-regulation of the *CBF2* pathway. In addition, the clock appeared to be rapidly reinitiated upon deacclimation. The responses of primary metabolism lagged behind transcriptional responses. However, both metabolites and transcripts approached the non-acclimated state after 24 h of deacclimation. TF genes transiently regulated during deacclimation are on one hand prime candidates as regulators of deacclimation, but they are also prime candidates for being clock regulated. Transient responses not related to circadian regulation were observed, indicating processes that may play an important role in the regulation of deacclimation.

## Additional files

Additional file 1: Relative expression values of 1462 TF genes analyzed in detail in this study. Data represent transcript levels determined by qRT-PCR expressed as 2^–ΔCt^. (XLSX 472 kb)

Additional file 2: Transcripts with significant up- or down-regulation relative to cold acclimated plants following 2 h, 4 h, 6 h, 12 h or 24 h of deacclimation (DEA) or in non-acclimated plants (NA). The listed values are the mean log_2_ fold changes calculated from expression data obtained by Affymetrix Genechip *Arabidopsis* Gene 1.0 ST Array microarray hybridization. (XLSX 369 kb)

Additional file 3: Metabolite data from GC-MS metabolome analysis normalized to FW and sorbitol. (XLSX 121 kb)

Additional file 4: Distribution of 476 significantly regulated genes encoding transcription factors among the clusters shown in Fig. 2. Major clusters in Fig. 2 are indicated by letters, while small cluster with only one or a few TF genes are indicated by numbers. (XLSX 33 kb)

Additional file 5: Scatterplot of the log_2_ relative expression values of 1462 transcription factor genes between cold acclimated and deacclimated and between cold acclimated and non-acclimated plants of *Arabidopsis thaliana*. The expression values were determined using qRT-PCR (TF platform) or the Affymetrix Genechip *Arabidopsis* Gene 1.0 ST Array (Microarray). (PDF 62 kb)

Additional file 6: PageMan analysis of coordinated changes of gene functional categories during 2 h, 4 h, 6 h, 12 h or 24 h of deacclimation (DEA) or in non-acclimated (NA) plants of *Arabidopsis thaliana* relative to cold acclimated plants. Normalized gene expression values were subjected to an overrepresentation analysis to identify functional categories that contained significantly more or less regulated genes than expected by chance. Blue color indicates significant enrichment of up- or down-regulated genes, red indicates significant depletion. (PDF 289 kb)

Additional file 7: Distribution of metabolites with significantly changed pool sizes among the clusters shown in Fig. 4. Major clusters in Fig. 4 are indicated by letters, while small cluster with only one or a few metabolites are indicated by numbers. (XLSX 11 kb)

Additional file 8: Transcription factor genes transiently up- or down-regulated upon transfer of cold acclimated plants to deacclimating conditions for 24 h (compare Fig. 2). Transiently regulated TF genes were identified using manual inspection and pair-wise t-tests. Genes highlighted in bold have been shown not to cycle under warm conditions. *P*-values are shown both before and after FDR correction. (XLSX 139 kb)

Additional file 9: Metabolites transiently up- or down-regulated upon transfer of cold acclimated plants to deacclimating conditions for 24 h (compare Fig. 4). Transiently regulated metabolites were identified using manual inspection and pair-wise t-tests. FDR corrected P-values are shown. (XLSX 17 kb)
