# Supplementary material for: Rapid transcriptional and metabolic regulation of the deacclimation process in cold acclimated Arabidopsis thaliana
Source: BMC Genomics. 2017 Sep 16;18:731. doi: 10.1186/s12864-017-4126-3 (PMC5602955; doi:10.1186/s12864-017-4126-3)
Supplement: Supplementary file 5 — Scatterplot of the log2 relative expression values of 1462 transcription factor genes between cold acclimated and deacclimated and between cold acclimated and non-acclimated plants of Arabidopsis thaliana. The expression values were determined using qRT-PCR (TF platform) or the Affymetrix Genechip Arabidopsis Gene 1.0 ST Array (Microarray). (PDF 62 kb) [file 12864_2017_4126_MOESM5_ESM.pdf]

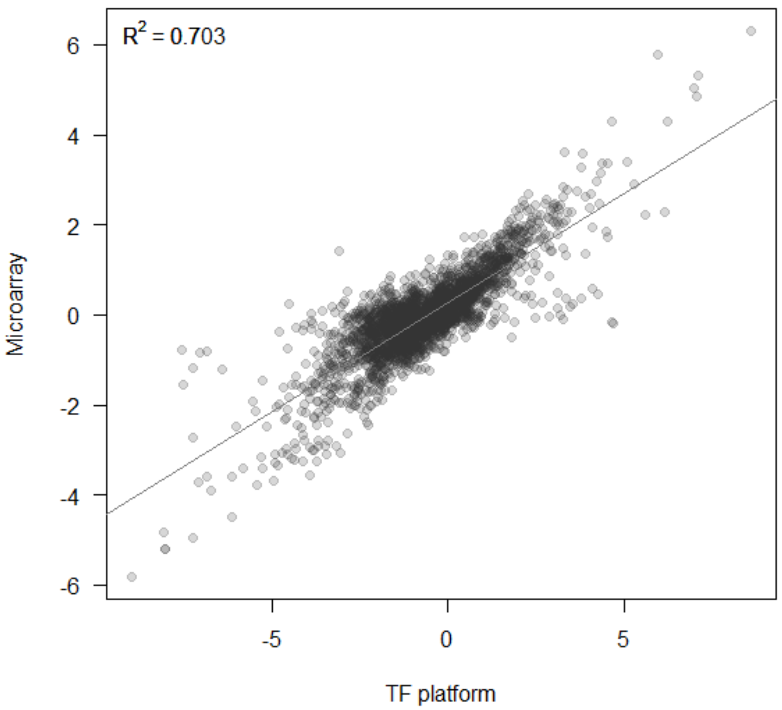

Additional file 5: Scatterplot of the $\log_2$ relative expression values of 1462 transcription factor genes between cold acclimated and deacclimated and between cold acclimated and non-acclimated plants of *Arabidopsis thaliana*. The expression values were determined using qRT-PCR (TF platform) or the Affymetrix Genechip *Arabidopsis* Gene 1.0 ST Array (Microarray).
