# Supplementary material for: Rapid transcriptional and metabolic regulation of the deacclimation process in cold acclimated Arabidopsis thaliana
Source: BMC Genomics. 2017 Sep 16;18:731. doi: 10.1186/s12864-017-4126-3 (PMC5602955; doi:10.1186/s12864-017-4126-3)
Supplement: Supplementary file 6 — PageMan analysis of coordinated changes of gene functional categories during 2 h, 4 h, 6 h, 12 h or 24 h of deacclimation (DEA) or in non-acclimated (NA) plants of Arabidopsis thaliana relative to cold acclimated plants. Normalized gene expression values were subjected to an overrepresentation analysis to identify functional categories that contained significantly more or less regulated genes than expected by chance. Blue color indicates significant enrichment of up- or down-regulated genes, red indicates significant depletion. (PDF 289 kb) [file 12864_2017_4126_MOESM6_ESM.pdf]

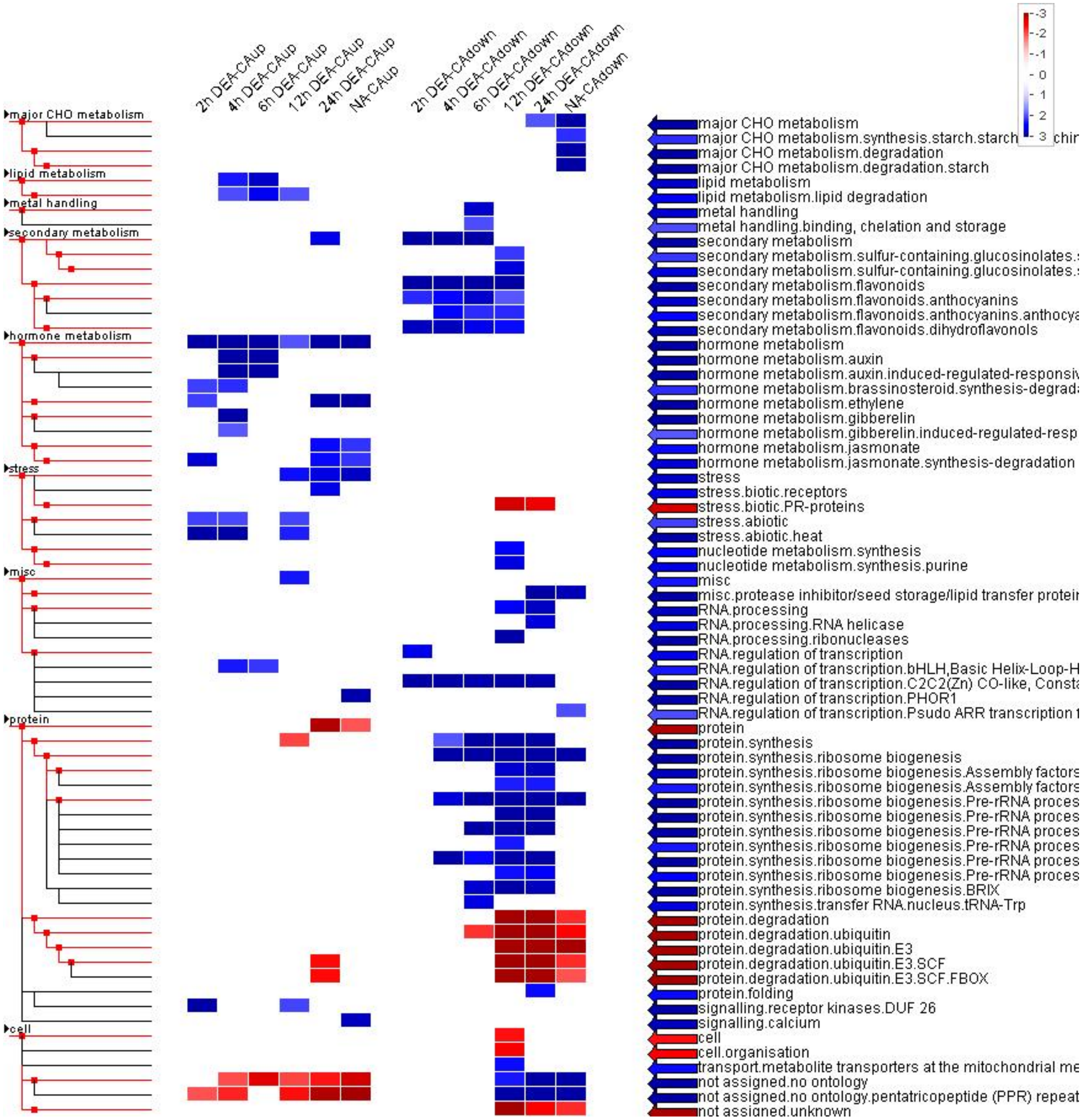

Additional file 6. PageMan analysis of coordinated changes of gene functional categories during 2 h, 4 h, 6 h, 12 h or 24 h of deacclimation (DEA) or in non-acclimated (NA) plants of *Arabidopsis thaliana* relative to cold acclimated plants. Normalized gene expression values were subjected to an overrepresentation analysis to identify functional categories that contained significantly more or less regulated genes than expected by chance. Blue color indicates significant enrichment of up- or down-regulated genes, red indicates significant depletion.
